# MiR-122 Inhibits Cell Proliferation and Tumorigenesis of Breast Cancer by Targeting IGF1R

**DOI:** 10.1371/journal.pone.0047053

**Published:** 2012-10-08

**Authors:** Biyun Wang, Hong Wang, Ziang Yang

**Affiliations:** 1 Department of Medical Oncology, Fudan University Shanghai Cancer Center, Department of Oncology, Shanghai Medical College, Fudan University, Shanghai, China; 2 Department of General Surgery, Zhongshan Hospital, Shanghai Medical College, Fudan University, Shanghai, China; National Institutes of Health, United States of America

## Abstract

miRNAs are emerging as critical regulators in carcinogenesis and tumor progression. Recently, microRNA-122 (miR-122) has been proved to play an important role in hepatocellular carcinoma, but its functions in the context of breast cancer (BC) remain unknown. In this study, we report that miR-122 is commonly downregulated in BC specimens and BC cell lines with important functional consequences. Overexpression of miR-122 not only dramatically suppressed cell proliferation, colony formation by inducing G1-phase cell-cycle arrest *in vitro*, but also reduced tumorigenicity *in vivo*. We then screened and identified a novel miR-122 target, insulin-like growth factor 1 receptor (IGF1R), and it was further confirmed by luciferase assay. Overexpression of miR-122 would specifically and markedly reduce its expression. Similar to the restoring miR-122 expression, IGF1R downregulation suppressed cell growth and cell-cycle progression, whereas IGF1R overexpression rescued the suppressive effect of miR-122. To identify the mechanisms, we investigated the Akt/mTOR/p70S6K pathway and found that the expression of Akt, mTOR and p70S6K were suppressed, whereas re-expression of IGF1R which did not contain the 3′UTR totally reversed the inhibition of Akt/mTOR/p70S6K signal pathway profile. We also identified a novel, putative miR-122 target gene, PI3CG, a member of PI3K family, which further suggests miR-122 may be a key regulator of the PI3K/Akt pathway. In clinical specimens, IGF1R was widely overexpressed and its mRNA levels were inversely correlated with miR-122 expression. Taken together, our results demonstrate that miR-122 functions as a tumor suppressor and plays an important role in inhibiting the tumorigenesis through targeting IGF1R and regulating PI3K/Akt/mTOR/p70S6K pathway. Given these, miR-122 may serve as a novel therapeutic or diagnostic/prognostic-target for treating BC.

## Introduction

Breast cancer (BC) is one of the leading causes of cancer deaths worldwide and the most common cancer among women [1]. It is a heterogeneous disease due to complicated etiology involving both genetic and environmental factors. Despite advances in treatment strategies, BC remains incurable and the goals of therapy range from symptom palliation to extending survival [2]. Given this, there is an urgent need to develop novel strategies for the diagnosis, treatment and prognosis of BC.

miRNAs are a class of small, endogenous, non-coding, single-stranded RNAs that regulate target-gene expression at post-transcriptional levels [3], [4]. They are considered to be part of a network wherein a modest change in one miRNA expression will set off a chain reaction involving multiple genes of the same or different pathways [5]. Currently, more than 1000 human miRNAs have been identified, and they could potentially modulate close to one-third of the coding genes in human genome [6]. miRNAs are involved in a range of processes that includes development, differentiation [7], proliferation, and apoptosis [8] and have been implicated in cancer [9]–[11]. Recent evidence has shown that about half of the human miRNAs are located in cancer-associated genomic regions that are frequently amplified, deleted, or rearranged in cancer, suggesting that some miRNAs may act as as tumor suppressor genes or oncogenes [9]–[11]. To date, several human miRNAs have been shown to be dysregulated in BC, such as miR-9, miR-125b, miR-301, miR-155, miR-135a and miR-122 [12]–[16], which contribute to the development and progression of BC. These findings suggest the involvement of miRNAs in BC tumorigenesis.

miR-122 is one of the most frequent miRNA isolated in the liver and plays important roles in many aspects of liver physiology, such as stress response [17] and lipid metabolism [18], [19]. Recently, Shoumei Bai et al. [20] found that miR-122 is frequently suppressed in primary hepatocellular carcinomas (HCCs), and can supress ADAM10 and SRF expression, which play key roles in tumorigenesis in various cancers. Leina Ma et al. [21] showed that miR-122 overexpression could induce apoptosis and cell cycle arrest in cancer cells through reducing the expression of Bcl-W and/or CCNG1. These findings suggest that miR-122 may behave as a tumour suppressor in human cancers. However, the biological function and mechanisms of miR-122 in breast cancer remain to be further elucidated.

In this study, we investigated the potential involvement of miR-122 in BC. We examined the expression of miR-122 in human BC cells and specimens and tested its effects on cell proliferation, cell-cycle distribution, and colony formation. In addition, we investigated a potential role of miR-122 on BC tumorigenesis in a murine model. Finally, we explored the underlying mechanism of miR-122 functions in BC. Our study will provide a better understanding of BC pathogenesis.

**Figure 1:**
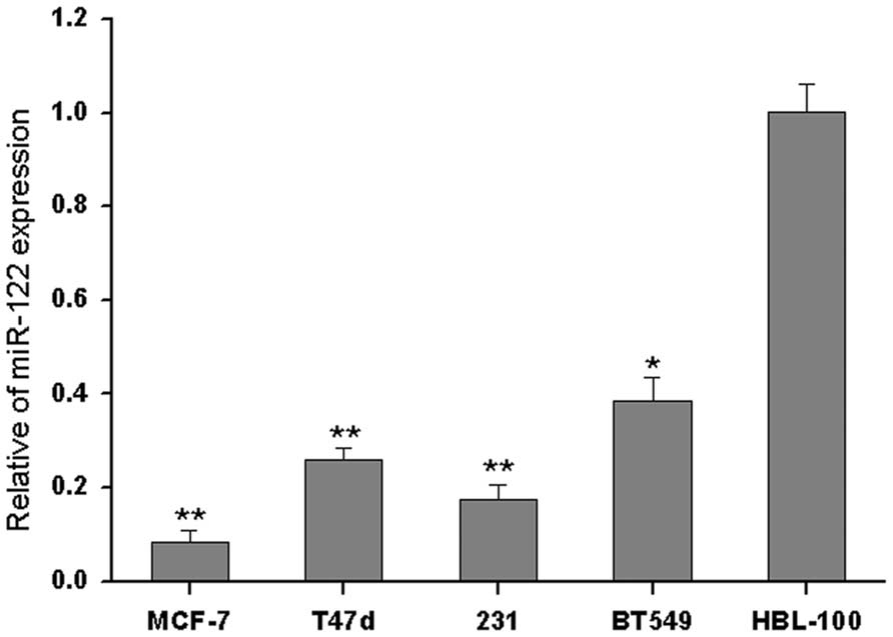
The expression of miR-122 is suppressed in BC cell lines. The miR-122 expression levels of MCF-7, T47d, MDA-MB-231, BT549 and HBL-100 cells were detected by qRT-PCR. The relative expression of miR-122 was normalized to the endogenous control U6. Each sample was analyzed in triplicate. (*p<0.05, **p<0.01).

## Materials and Methods

### Cell Lines and Cell Culture

MCF-7, T47d, MDA-MB-231, BT549 and HBL-100 cell lines were provided by Institute of Biochemistry and Cell Biology of Chinese Academy of Science (China) and originated from ATCC. The cells were cultured in Dulbecco’s modified Eagle’s medium (DMEM) supplemented with 10% fetal bovine serum, 2 µM glutamine, 100 IU/ml penicillin, and 100 µg/ml streptomycin sulfate. All cell lines were incubated at 37°C under a 5% CO_2_ condition.

**Figure 2:**
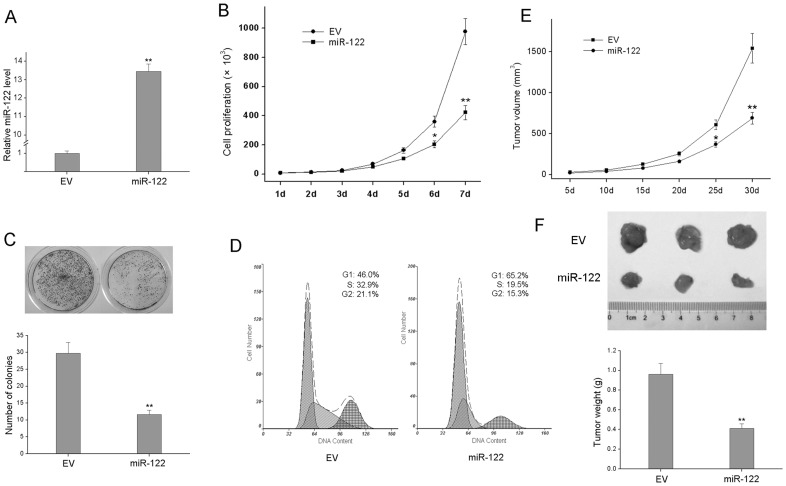
Overexpression of miR-122 suppresses cell proliferation of MCF-7 cells in vitro and in vivo. (A) miR-122 was over-expressed in MCF-7 cells and confirmed by qRT-PCR. (B) Growth curves of miR-122 and EV-infected MCF-7 cells were conducted. (C) Colony formation was assayed in miR-122 and EV-infected cells, and colonies consisting more than 50 cells were counted. (D) Cell cycle progression was assayed using flow cytometric analysis. (E) Tumor size observation in nude mice after the inoculation. The average size of the tumors was measured every 5 days and shown in the curves. (F) Tumor sizes of 3 representative nude mice. The tumor weight was measured. All experiments were carried 3 times independently. *P<0.05 compared to EV group. **P<0.01 compared to EV group.

### Vector Constructs

To construct IGF1R-3′UTR plasmid, a wild-type 3′-UTR fragment of human IGF1R mRNA (Genbank accession no.NM_000875) containing the putative miR-122 binding sequence was amplified by PCR and cloned into the site between *Xba*I and *Fse*I of the pGL3-control vector (Promega, USA) which is the downstream of the luciferase reporter gene. The corresponding mutant constructs were created by mutating the seed regions of the miR-122-binding sites (5′-CACUCCA-3′ to 5′-CAgaCCA-3′). The nucleotide sequences of primers for IGF1R-3′UTR clone were: 5′-gctctagagcCCACTGTTGATGCAGGTTTG-3′ (forward) and 5′-tataggccggcctaAAAACAACTAAAGGGGCAGG-3′ (reverse) and the mutagenesis primers were: 5′-CCCCTTTCTGCTCACTCCAAGAA-3′ (forward) and 5′-TTCTTGGAGTGAGCAGAAAGGGG-3′ (reverse).

**Figure 3:**
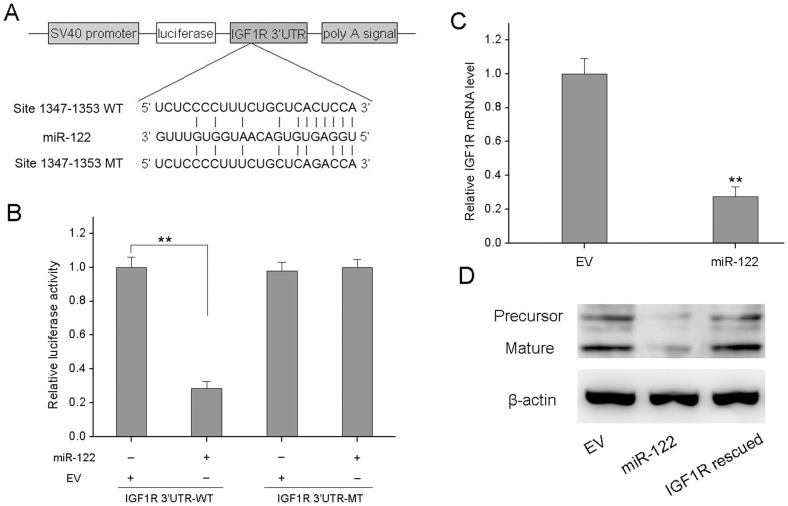
IGF1R is directly targeted by miR-122. (A) IGF1R 3′UTR and IGF1R 3′UTR-MT were inserted into the region immediately downstream of the luciferase gene in pGL3 vector. (B) Dual luciferase reporter assays were performed. The expression of the reporter containing IGF1R 3′UTR was suppressed by miR-122, but not in the mutated construct. qRT–PCR (C) and Western blot (D) analyses were performed to examine the effects of miR-122 on IGF1R expression in MCF-7 cells. Both precursor and mature IGF1R (2 bands) were suppressed after miR-122 overexpression. The suppression was abolished by transfection of the cells with IGF1R cDNA without 3′UTR. All experiments were carried 3 times independently. *P<0.05 compared to EV group. **P<0.01 compared to EV group.

### Lentiviral Vector Production and Infection

To construct the lentivirus vector Lv-miR-122 which over-expressed miR-122, a fragment encoding the pre-miR-122 sequence plus 200 bp at both 5′- and 3′-flanking regions was amplified with the primers 5′-CCGACGCGTCCGTGATGCTTCTTTTCTCT-3′ (forward) and 5′-CCGGCG GCCGCTGAGTGCAAAAGAGCCAGAC-3′ (reverse) by PCR from human genomic DNA and then cloned into the *Mlu* I/*Not* I sites of pLemirR vector. Then, 293T cells were co-transfected with the four vector plasmids: pLemirR-miR-122, pRsv-REV, pMDlg-pRRE, and pMD2G. For lentiviral infection, cells were plated at a concentration of 1×10^6^ cells in 25 mL flask to reach 60–80% confluence after overnight culture and were then infected at MOI of 20 in the presence of polybrene (8 µg/ml). Positively infected cells were sorted by FACS to be continued to culture. The level of miR-122 was detected by realtime PCR. Another two lentiviral vectors for cDNA and shRNA delivery of IGF1R were described previously [22].

**Figure 4:**
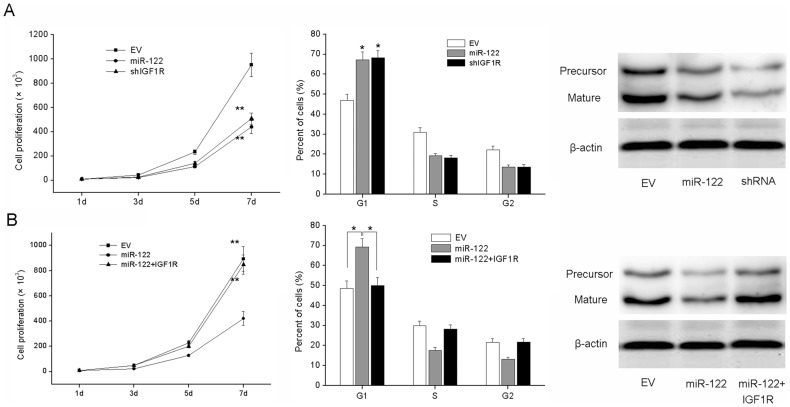
IGF1R is involved in miR-122-induced growth inhibition in BC cells. (A) MCF-7 cells were infected with Lv-shIGF1R or Lv-miR122. Cell growth rate and cell-cycle distribution were measured. (B) MCF-7 cells were infected with LV-miR122 for 72 hours, followed by infection with LV-IGF1R, and cell proliferation and cell-cycle analysis were then performed. *P<0.05 compared to EV group or comparison between 2 groups as indicated, **P<0.01 compared to EV group or comparison between 2 groups as indicated.

### Cell Proliferation and Cell Cycle Analysis

For analysis of cell proliferation, cells were seeded onto 24-well plates at 5×10^3^ cells/well and the cell numbers were determined daily for one week. For analysis of cell cycle, cells were suspended in 1 ml solution containing 0.4 mM sodium citrate, 25 µg/ml propidium iodide (PI), and 50 µg/ml RNase. The stained cells were analyzed in a FACScan ﬂow cytometer (BD Biosciences, USA) using the ModFit LT program (BD Biosciences, USA).

**Figure 5:**
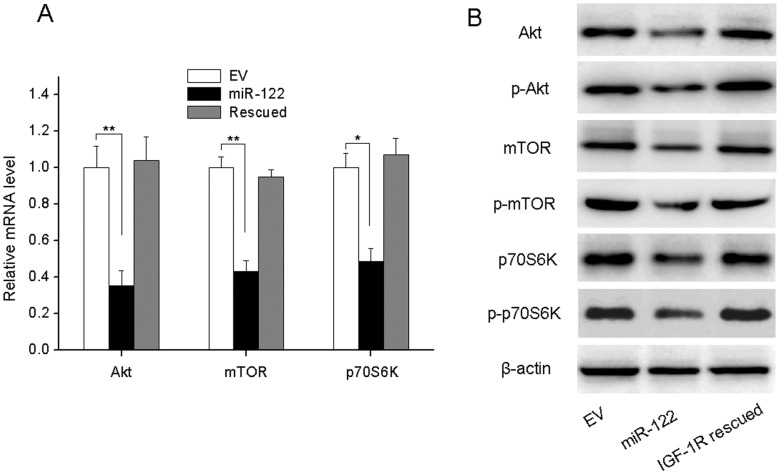
Overexpression of miR-122 alters the expression of key components in the PI3K/Akt/mTOR/p70S6K signal pathway. (A) miR-122-overexpression significantly suppressed the PI3K/Akt/mTOR/p70S6K pathway examined by qRT–PCR (A) and Western blot (B). The inhibition of PI3K/Akt/mTOR/p70S6K was reversed by re-expression of IGF1R (IGF1R rescued) in miR-122 group. All experiments were carried 3 times independently. *P<0.05 compared to EV group. **P<0.01 compared to EV group.

### Colony Formation Assay

The assay was conducted as previously described [23]. Briefly, cells were digested with trypsin and suspended into a single cell status. 5000 cells from each group were cultured in the 60 mm diameter culture dish with 10% FBS for 14 days. The colonies were fixed and stained with 0.5% crystal violet for 15 min, and then washed 3 times. Colonies less than 2 mm in diameter and faintly stained were ignored. The number of colonies in 10 random view fields was counted under a microscope and the average representing the 95% confident region was achieved. The experiment was carried out 3 independent times.

**Figure 6:**
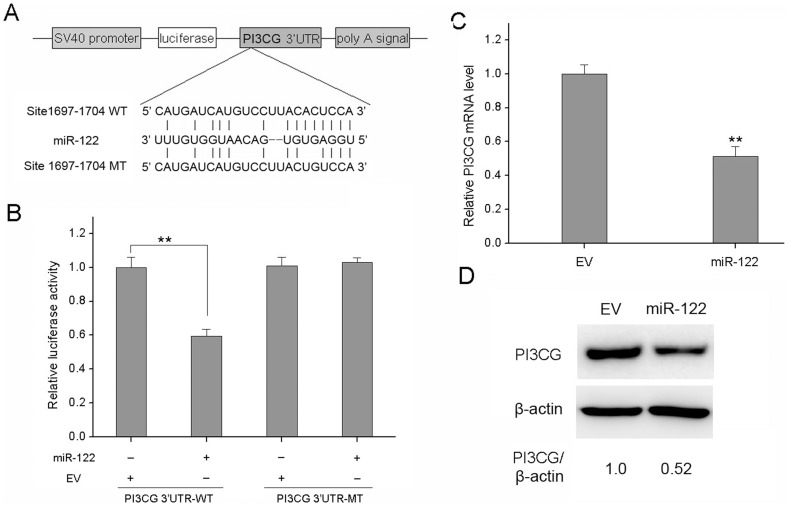
miR-122 down-regulates PI3CG expression by directly binding to its mRNA 3′UTR. (A) PI3CG 3′UTR and PI3CG 3′UTR-MT were inserted into the region immediately downstream of the luciferase gene in pGL3 vector. (B) Dual luciferase reporter assays were performed. The expression of the reporter containing PI3CG 3′UTR was suppressed by miR-122, but not in the mutated construct. qRT–PCR (C) and Western blot (D) analyses were performed to examine the effects of miR-122 on PI3CG expression in MCF-7 cells. All experiments were carried 3 times independently. *P<0.05 compared to EV group. **P<0.01 compared to EV group.

### RNA Extraction and Quantitative RT-PCR

Total RNA was extracted from cell lines using Trizol reagent (Invitrogen, Carlsbad, CA, USA) and subsequently treated with RNase-free DNase I (Fermentas, San Diego, CA, USA). For qRT-PCR analysis of IGF1R, Akt, mTOR, p70S6K, PI3CG and β-actin mRNA expression, 1 µg of total RNA was reverse transcribed to cDNA with random hexamers and Thermoscript (Invitrogen). Real-time PCR was performed using an ABI PRISM 7900 Sequence Detection System (Applied Biosystems) with Quanti-Tect SYBR Green PCR mixture (Qiagen). The sets of primers were for IGF1R: forward 5′-GGACAGGTCAGAGGGTTTC-3′, reverse 5′-CTCGTAACTCTTCTCTGTGCC-3′; for Akt: forward 5′-TTTGTCATGGAGTACGCCAATG-3′, reverse 5′-CACAATCTCCGCACCGTAGAA-3′; for mTOR: forward 5′-CGCTGTCATCCCTTTATCG-3′, reverse 5′-ATGCTCAAACACCTCCACC-3′; for p70S6K: forward 5′-TTATGTTCTTTTTCCCCCTT-3′, reverse 5′-TGCCCACAAATTATCTTCTATC-3′; for PI3CG: forward 5′-ATGGAGCTGGAGAACTATAAAC-3′, reverse 5′-TTCCGCGGCCTCCTACTTCGGCG-3′; and for β-actin: forward 5′-GAGCTACGAGCTGCCTGACG-3′, reverse 5′-CCTAGAAGCATTTGCGGTGG-3′. The relative expression level was determined using the 2^−ΔΔCt^ analysis method, where β-actin was used as an internal reference.

**Figure 7:**
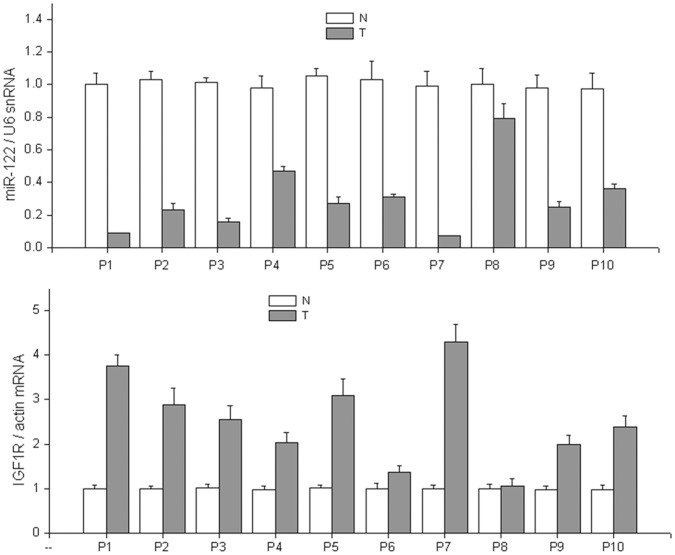
Repression of miR-122 correlates with the upregulation of IGF1R. The expression of miR-122 and IGF1R was detected by qRT-PCR in 10 pairs of BC samples. miR-122 was found to be repressed in all BCs. P, Patient; N, Normal tissue; T, BC tumor.

**Figure 8:**
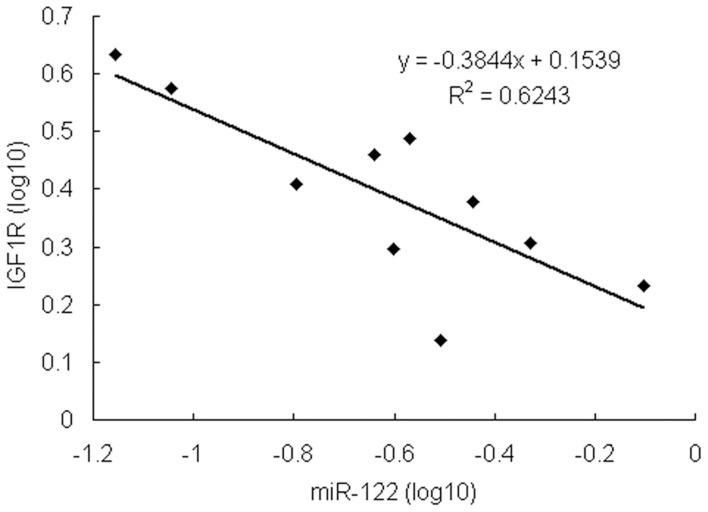
The expression of miR-122 and IGF1R is negatively correlated in clinical BC tissues. There may be an inverse relationship between the expression of miR-122 and IGF1R.

For analysis of miR-122 expression by qRT-PCR, 50 nM miRNA specific stem-loop RT primers [24] were used for reverse transcription reaction as the following. 5′- TCTTCCTGGAATTCAAGCCTTT-3′ and 5′-AGTGGGCCTAGTGCTGGAAA -3′ for miR-122; 5′-CTCGCTTCGGCAGCACA-3′ and 5′-AACGCTTCACGAATTTGCGT -3′ for RNU6. PCR was performed by ABI PRISM 7900 Sequence Detection System. All reactions were run in triplicate and all experiments were carried 3 independent times.

### Western Blot

Cell extracts were prepared in ice-cold RIPA lysis buffer. After whole cell protein extracts were quantified by BCA protein assay, equivalent amounts of cell lysates were resolved by 10% SDS polyacrylamide gel electrophoresis and transferred onto a polyvinylidene diﬂuoride membrane, which was then blocked in 5% non-fat milk in PBST for 1 hour at 4°C. The blots were then incubated with primary antibodies against IGF1R, Akt, p-Akt (Ser473), mTOR, p-mTOR (Ser2448), p70S6K, p-p70S6K (Thr444), PI3CG and β-actin (Abcom, Cambridge, UK). After incubation with horseradish peroxidase-conjugated secondary antibody, protein bands were visualized using enhanced chemilu-minescence detection kit (Millipore, Billerico, Massochusatts, USA). The intensity of the bands was analyzed using Image-Pro Plus software.

### Luciferase Reporter Assay

MCF-miR-122 and MCF-EV cells were transfected with IGF1R 3′UTR plasmid and Renilla luciferase pRL-TK vector (Promega USA) using lipofectamine 2000 reagent (Invitrogen, USA) according to the manufacturer’s protocol. Luminescence was assayed 48 hours later using the Dual-Luciferase Reporter Assay System (Promega, USA) according to the manufacturer’s instructions. Results were normalized to the Renilla luminescence from the same vector and shown as the ratio between the various treatments and cells transfected with control vector.

### Tumor Growth Assay

Female BALB/c nude mice aged 4 to 5 weeks were housed and manipulated according to the protocols approved by Shanghai Medical Experimental Animal Care Commission. For tumor growth assay, 5×10^6^ “MCF-EV” and “MCF-miR-122” cells were injected subcutaneously into the left and right scapulas respectively in 100 µl of serum-free medium. Tumor growth was measured every 5 days for 30 days.

### Clinical BC Sample Analysis

Ten surgical specimens (both tumor and adjacent normal tissue) were obtained from patients in Zhongshan Hospital (Fudan University, Shanghai, China) and were snap-frozen in liquid nitrogen and stored at −80°C for later RNA extraction. Informed consent was obtained from each patient, and the research protocols were approved by the Committee for Ethical Review of Research Involving Human Subjects at Fudan University.

### Statistical Analysis

SPSS 13.0 software was used for statistical analysis. Data were presented as mean ± SD of at least 3 independent experiments. Two-tailed t test was used for comparisons of 2 independent groups. The relationship between IGF1R and miR-122 expression was explored using Pearson χ2 test. Data were considered to be statistically significant when p<0.05 (*) and p<0.01 (**).

## Results

### miR-122 is Downregulated in Human BC Cells

To investigate the biological role of miR-122 in BC cells, we first examined the expression of miR-122 in BC cell lines including MCF-7, T47d, MDA-MB-231, and BT549 cells. As shown in Figure 1, miR-122 showed significantly lower expression in human BC cells compared to human breast epithelial cell line HBL-100, suggesting that the downregulation of miR-122 may be involved in breast carcinogenesis.

### miR-122 Overexpression Inhibits BC Cell Proliferation in vitro and in vivo

To study the role of miR-122 in BC, we established a miR-122 over-expression model in MCF-7 cells infected with miR-122 by Lentivirus pLemirR system, and cells infected with empty virus vector (EV) was used as a control. The expression of miR-122 in miR-122 group was upregulated 13.5 folds compared to EV group, which was confirmed by qRT-PCR (Figure 2A). Using cell proliferation assay, we observed a significant decrease in cell number in MCF-miR-122 cells versus MCF-EV cells on day 7 (Figure 2B). Similarly, miR-122 overexpression resulted in decreased colony formation and marked G1 cell-cycle arrest compared to EV group (Figure 2C, D).

To further confirm the above findings, 5×10^6^ EV or miR-122-infected cells were inoculated subcutaneously into the right and the left scapula of each mouse respectively (n = 3). Though tumors formed in scapulas inoculated with either MCF-EV or MCF-miR-122 cells, the tumor growth of the MCF-miR-122-induced tumors was significantly reduced (Figure 2E). The average tumor weight of the MCF-EV group was 2.54 folds higher than that of the MCF-miR-122 group after 30 days post-inoculation (Figure 2F). These results showed that miR-122 inhibits the proliferation of MCF-7 cells both *in vitro* and *in vivo*.

### IGF1R is a Direct Target of miR-122 in BC Cells

Next, we wanted to investigate the mechanism of miR-122 inhibition of proliferation of MCF-7 cells. We tried to search for the target genes of miR-122 using TargetScan. Among the predicted miR-122 targets, IGF1R was found to be a likely target because it contains putative miR-122 target sites in its 3′UTR (Figure 3A). To directly test whether IGF1R was targeted by miR-122, we cloned the 3′UTR of IGF1R into a reporter plasmid downstream from luciferase and performed reporter assays (Figure 3A). We observed that the relative luciferase activity of the reporter which contained wild-type 3′UTR of IGF1R was significantly inhibited in miR-122 group compared to EV group. However, the relative luciferase activity of the mutant IGF1R 3′UTR reporter was almost at the same level as the control group and failed to respond to miR-122 (Figure 3B).

To assess whether miR-122 had a functional role in downregulation of endogenous IGF1R expression, the IGF1R expression was determined using qRT-PCR and Western blot. The result showed that IGF1R mRNA was reduced significantly in miR-122 group compared to EV group (Figure 3C). IGF1R protein levels were also significantly suppressed in miR-122 group (Figure 3D). Transfection with IGF1R cDNA could totally overcome the suppression caused by miR-122 (Figure 3C, D) because the construct contained no 3′UTR. Taken together, our data identified IGF1R as a direct miR-122 target and suggested that miR-122 overexpression suppresses the expression of IGF1R in MCF-7 cells.

To elucidate whether the growth-suppressive effect of miR-122 was mediated by down-regulation of IGF1R in BC cells, we performed gain-of-function and loss-of-function studies. First, we silenced IGF1R to investigate whether the reduced expression of IGF1R could mimic the suppressive effect of miR-122. MCF-7 cells were infected with shIGF1R or EV and then we examined cell growth rate and cell cycle distribution. As shown in Figure 4A, knockdown of IGF1R led to significant cell growth inhibition and cell-cycle arrest, which is similar to those induced by miR-122. Next, we evaluated whether ectopic expression of IGF1R could rescue the suppressive effect of miR-122. MCF-7 cells were infected with Lv-miR122 for 72 hours and followed by transfection with IGF1R cDNA. We showed that ectopic expression of IGF1R significantly rescued miR-122-induced cell growth inhibition and cell-cycle arrest (Figure 4B).

### miR-122 Regulates the Expression of Key Components of the PI3K/Akt Signaling Pathway in BC Cells

We then sought to determine whether the IGF1R-mediated downstream signal pathway was also impacted by miR-122. To this goal, we examined the expression of key components of the PI3K/Akt signaling pathway. We found that the transcription of Akt, mTOR and P70S6K, major components of the PI3K/Akt pathway, was significantly down-regulated in MCF-miR-122 cells (Figure 5A). The total and phosphorylated protein levels of all three molecules described above showed the same results (Figure 5B). Moreover, re-expression of IGF1R could totally reverse the inhibition of PI3K/Akt signal pathway profile (Figure 5A, B). These results indicated that miR-122 may be an important regulator of this signaling pathway.

Since one miRNA could regulate multiple and functionally related targets in one pathway, we wondered whether there are additional mRNA targets that may be regulated. As a result, we identified PI3CG (p110γ) as a putative target of miR-122 (Figure 6A) and indeed observed that it was targeted by miR-122 at both mRNA and protein levels (Figure 6C, D). Using luciferase reporter assay, we found that the relative luciferase activity of the reporter which contained the putative miR-122 target site was significantly reduced in miR-122 group (Figure 6B). These data suggested that miR-122 can inhibit BC cell proliferation by regulating the PI3K/Akt signaling pathway.

### miR-122 Regulates IGF1R Expression in Clinical BC Specimens

To further investigate whether miR-122 is involved in BC progression through the regulation of IGF1R expression, the expression of miR-122 and IGF1R were analyzed by qRT-PCR in 10 pairs of clinical BC samples (Figure 7). Compared to paired normal tissues, repression of miR-122 expression was detected in all BC cases. In accordance with the miR-122 repression, the expression of IGF1R was upregulated in 9 out of 10 BC cases. Correlation analysis indicated that IGF1R expression was reduced along with miR-122 overexpression in these 10 pairs of HCC specimens (r^2^ = 0.624, Pearson χ^2^ test, Figure 8).

## Discussion

In the last decade, miRNAs have emerged as critical regulators in cancer-related processes [25], [26]. Studies have shown that tumor-targeting therapies using miRNAs is becoming a novel diagnostic and therapeutic tool [27]. In this study, we focused on miR-122, which has been demonstrated to suppress tumor growth in liver cancers [20]. Jelena Radojicic and colleagues recently found that miR-122 exhibited significantly lower expression in BC cases compared to the normal tissue [28]. This result was consistent with the reduction of miR-122 level in BC cell lines and BC specimens in our study. Furthermore, we observed that overexpression of miR-122 inhibits cell growth, colony formation in vitro, and the tumorigenesis in vivo. These results indicate that miR-122 may function as a negative regulator or tumor suppressor for the cell growth, which is consistent with the role of miR-122 in HCC [29].

To date, several targets of miR-122 have been identified, such as ADAM10, SRF, MAP3K12, Ndrg3, AldoA, Bckdk and CD320 [20], [30]. On the basis of bioinformatics analysis, we further predicted several additional miR-122 targets, including IGF1R. Our present experimental results confirm that IGF1R, as well as PI3CG, are functional targets of miR-122 in BC cells. There are several lines of evidence to support this. Firstly, miR-122 overexpression significantly down-regulated IGF1R by directly targeting the 3′UTR of IGF1R mRNA confirmed using luciferase-reporter-gene assays. This effect was largely eliminated when the sites in IGF1R 3′UTR targeted by miR-122 were mutated. Moreover, both mRNA and protein of IGF1R were significantly decreased in miR-122 group compared to EV group. However, overexpression of IGF1R without 3′UTR could overcome the inhibition by miR-122 and rescue the expression of IGF1R. These results strongly suggested that miR-122 suppresses the expression of IGF1R through directly interacting with the wild type 3′UTR of it.

IGF1R is a receptor tyrosine kinase which consists of heterotetramers (α2β2) held together by disulfide bridges, and it mediates IGF1-induced signaling events. IGF signaling abnormality appears to directly interfere with the normal cell growth regulation and proapoptotic responses triggered by activation of p53, the tumor suppressor, upon the treatment with anti-cancer agents [31]. In this study, we clearly demonstrate that PI3K/Akt/mTOR/p70S6K signal pathway is suppressed by miR-122. We also observed that re-expression of IGF1R in miR-122 group could overcome the inhibition effect of miR-122 and reversed the inhibition of IGF1R-mediated downstream Akt/mTOR/p70S6K signal pathway. It is worth noting that PIK3CG, one of the PI3K members, is also predicted by bioinformatics analysis to be a potential target of miR-122. Our luciferase-reporter-gene assays suggested that the relative luciferase activity was significantly reduced for the reporter plasmid contained the putative miR-122 target site but not the corresponding mutant counterpart was co-transfected with miR-122. Therefore, miR-122 concurrently targets at least two major signaling molecules in the IGF1R/PI3K/Akt/mTOR/p70S6K signaling pathway in BC.

In conclusion, our results indicate that miR-122 co-ordinately regulates IGF1R signaling at multiple levels. As a tumor suppressor in BC, overexpression of miR-122 inhibits BC cell growth both in vitro and in vivo. Our results show that miR-122 regulates cell proliferation through the PI3K/Akt/mTOR/p70S6K signaling pathway and exogenous overexpression of miR-122 may represent a promising approach for targeted BC therapies.
